# Genetic polymorphisms of antioxidant enzymes CAT and SOD affect the outcome of clinical, biochemical, and anthropometric variables in people with obesity under a dietary intervention

**DOI:** 10.1186/s12263-017-0590-2

**Published:** 2018-01-08

**Authors:** César Hernández-Guerrero, Alicia Parra-Carriedo, Diana Ruiz-de-Santiago, Oscar Galicia-Castillo, Mario Buenrostro-Jáuregui, Carmen Díaz-Gutiérrez

**Affiliations:** 1 Departamento de Salud, Universidad Iberoamericana, Prol. Paseo de la Reforma 880, Col Santa Fe, 01219 México City, Mexico; 2 Especialidad en Obesidad y Comorbilidades, Universidad Iberoamericana, Ciudad de México, Mexico; 3 Departamento de Psicología, Universidad Iberoamericana, Ciudad de México, Mexico

**Keywords:** Obesity, Genetic polymorphism, SOD, GPX, CAT

## Abstract

**Background:**

Genetic polymorphisms of antioxidant enzymes CAT, GPX, and SOD are involved in the etiology of obesity and its principal comorbidities. The aim of the present study was to analyze the effect of aforementioned SNPs over the output of several variables in people with obesity after a nutritional intervention. The study included 92 Mexican women, which received a dietary intervention by 3 months. Participants were genotyped and stratified into two groups: (1) carriers; mutated homozygous plus heterozygous (CR) and (2) homozygous wild type (WT). A comparison between CR and WT was done in clinical (CV), biochemical (BV), and anthropometric variables (AV), at the beginning and at the end of the intervention.

**Results:**

Participants (*n* = 92) showed statistically significant differences (*p* < 0.05) at the end of the nutritional intervention in several CV, BV, and AV. However, two kinds of responses were observed after genotyping participants: (A) CR and WT showed statistically significant differences (*p* < 0.05) in several CV, BV, and AV for the SNPs 599C>T GPX1 (rs1050450), − 251A>G SOD1 (rs2070424), and − 262C>T CAT (rs1001179). (B) Only CR showed statistically changes (*p* < 0.05) in several CV, BV, and AV for the SNPs − 21A>T CAT (rs7943316) and 47C>T SOD2 (rs4880). The dietary intervention effect was statistically significantly between the polymorphisms of 47C>T SOD2 and BMI, SBP, TBARS, total cholesterol, and C-LCL (*p* < 0.05) and between the polymorphisms of − 21A>T CAT (rs7943316) and SBP, DBP, total cholesterol, and atherogenic index (*p* < 0.05).

**Conclusion:**

People with obesity display different response in several CV, BV, and AV after a nutritional intervention, depending on the antioxidant genetic background of SOD and CAT enzymes.

## Background

Due to changes in lifestyle and eating behaviors of people, the worldwide prevalence of overweight and obesity has increased dramatically in the last decades [1]. The presence of obesity in the population has increased the risk to develop insulin resistance, cardiovascular diseases, metabolic syndrome, type II diabetes, dyslipidemia, cancer, and others [2, 3]. If an important decrement in the prevalence of overweight and obesity is not achieved, the medical care for such pathologies will far exceed the technical and economic capacity of the health systems in the following years [2–4].

The multifactorial etiology of obesity involves a complex interaction between environment, feeding, physical activity, culture, and genetic factors [5, 6]. Until now, the initial strategy to control the obesogenic process in a person is to reduce the caloric intake and to increase the energetic expenditure by means of physical activity. However, due to genetic background of diverse single-nucleotide polymorphisms (SNPs), evidence in the literature has shown a different response for people with obesity or associated obesity diseases under a caloric restriction program to modify body composition [7], peripheral lipid concentration [8], blood pressure [9], and insulin resistance [10].

In this sense, SNPs of antioxidant enzymes as catalase (− 262C>T CAT, rs1001179, and − 21A>TCAT, rs7943316), superoxide dismutase (− 251A>G SOD1, rs2070424, and 47C>T SOD2, rs4880), and glutathione peroxidase (599C>T GPX1, rs1050450) have been identified in coding and regulatory untranslated regions of the gene, which affect the net activity of the enzymes [11–14]. These enzymes work as a key factor to avoid the appearance of oxidative stress condition, inactivating the generation and propagation of endogenous free radicals produced by the cell metabolism [15].

The oxidative stress condition plays an important role in the etiology of the principal comorbidities of obesity. That condition appears when an imbalance of pro-oxidant systems overtakes the antioxidant defense. Diverse mechanisms increase the generation of free radicals in people with obesity as hyperleptinemia, endothelial reactive oxygen species production, chronic inflammation, elevated tissue lipid levels, hyperglycemia, and inadequate antioxidants intake [16, 17].

The genetic characteristics of people have been identified as a pivotal factor involved in the etiology of metabolic pathologies as obesity. Several SNPs of CAT, GPX, and SOD have been associated with obesity and its principal comorbidities [18–25]. Our research group previously identified an association with obesity for the SNPs 599C>T GPX1 (rs1050450) and − 251A>G SOD1 (rs2070424). For the SNPs − 21A>T CAT (rs7943316), − 262C>T CAT (rs1001179), and 47C>T SOD2 (rs4880), we identified differences between people with obesity CR (homozygous plus heterozygous) of mutated allele, and obese WT people, with respect to anthropometric and clinical variables in a cross-sectional study [26, 27].

The aim of the present work was to assess the impact of the five before mentioned SNPs of CAT, GPX, and SOD, with respect to changes on anthropometric, clinical, and biochemical variables, in a population with obesity after a nutritional intervention of energy restriction by 3 months, with the intention to identify candidate genes which could be included in the follow-up, evolution and prognosis of obesogenic process, and its main comorbidities.

## Methods

### Subjects and dietary intervention

The study included women with obesity who attended to the Nutrition Clinic of Universidad Iberoamericana in Mexico City to receive nutritional care. All subjects were from a low-income community from Mexico City. The inclusion criteria required a minimum age limit for participants of 18 and a maximum of 65 years of age, no history of eating disorders, thyroid disease, infectious disease, autoimmunity, allergies, non-smokers**,** and no history of consumption of antioxidant supplements within the past 6 months. Participants with previous diagnosis of diabetes, dyslipidemia, hypertension, or metabolic syndrome diagnostic were not included in the study.

Total energy intake was estimated by a food record method (24-h dietary recalls) over three non-consecutive days including a weekend or a holiday [28]. All the participants received an individual nutritional intervention that included an energy restriction of 500 kcal, with a nutrient distribution of 50% carbohydrates, 25% protein, and 25% fat with a fiber intake of at least 25 g/day, with the strict indication to walk at a “brisk pace” by 30 min per day. The participants enrolled in the study assisted the Nutrition Clinic every 2 weeks for a period of 3 months to check the progress of the dietary intervention and to receive coaching. They received a manual to record their adherence to the nutritional intervention and physical activity. Only participants with an adherence of at least 90% to the physical and nutritional intervention were included in the analysis.

### Determination of antioxidants consumption frequency and total energy expenditure

A food frequency questionnaire validated by the Mexican National Institute of Public Health was applied to assess the antioxidant intake of participants in the first visit to the Nutrition Clinic [29]. Indirect calorimetry method using a CardioCoach metabolic monitor 9002-CO2 (KORR Medical Technologies, Salt Lake City, Utah) was used to evaluate the resting metabolic rate. Total energy expenditure was predicted as RMR times physical activity level [30].

### Identification of polymorphisms of CAT, GPX, and SOD by PCR-RFLP method

The conditions, primers sequences, and identification of WT and mutated alleles of the five genetic polymorphisms have been described previously [26, 27].

### Determination of glucose, lipids, and thiobarbituric acid reactive substances (TBARS) in peripheral plasma

A sample of peripheral fasting blood was collected before and after the dietary intervention to assess the biochemical markers. The concentration of glucose (GC), total cholesterol (TC), LDL cholesterol (LDL-C), HDL cholesterol (HDL-C), and triglycerides (TG) was determined by Alere-Cholestec LDX System (Alere, San Diego, California). The lipid peroxidation state of peripheral plasma of each participant was determined by TBARS technique [31].

### Statistical analysis

Data were tested for normal distribution before statistical procedures were performed. Non-normally distributed data were log transformed to reduce skewness. Two-way ANOVA with replication was used to analyze the intervention effect between polymorphism and the dependent variables over the period of time (beginning and the end of the nutritional intervention)–two groups (WT and CR subjects) for each polymorphism–analyzing the interaction between period and group. Differences between the beginning and the end of nutritional intervention within groups were assessed by the paired *t* test for parametric data or Wilcoxon signed-rank test for non-parametric data. Differences between groups (WT and CR) before and after the intervention were compared using a two-sample *t* test test for parametric data or Mann-Whitney *U* test for non-parametric data.

In order to explore whether other variables could potentially affect the results of the study, multivariate analyses were carried out taking the variables related to the response of the dietary intervention as dependent variables, the SNPs showing a statistically significant interaction (observed in the two-way ANOVA with replication analysis) as independent variable and controlling for age, BMI, and the other SNPs of interest. Different models with adjustments were used for multivariate analyses: model 1 was adjusted for − 21A>T CAT (rs7943316) or 47C>T SOD2 (rs4880); model 2 was adjusted for age and BMI; model 3 was adjusted for − 21A>T CAT (rs7943316) or 47C>T SOD2 (rs4880), age, and BMI (variables in models 1 and 2); and model 4 was adjusted for variables in model 3 plus the other explored SNPs (599C>T GPX1 (rs1050450), − 251A>G SOD1 (rs2070424), and − 262C>T CAT (rs1001179). SPSS version 21 for Windows was used to do all the statistical analysis; a *p* < 0.05 was accepted as a significant statistical difference.

## Results

The study included 153 women with obesity with a mean age of 40 ± 13 years from a low-income community located in Mexico City at the beginning of the dietary intervention. However, only 92 women (age 40 ± 9.2) were included in the analysis of the data since 61 (39.8%) women did not have enough adherence to the trial (less than 90%).

With the intention of identifying if possible changes observed in subjects at the end of the study could be influenced by differences in feeding habits, consumption of antioxidants and pro-oxidants was assessed during the first visit to the Nutrition Clinic. Participants were stratified into two groups by their genotype. One group was constituted by CR of the mutated allele (homozygous plus heterozygous), and the other group was of individuals homozygous for the WT allele; this classification was done by each one of the five SNP studied (data not shown).

The results showed very few differences between the consumption of antioxidants (zinc, copper, manganese, selenium, and vitamins A, C, and E) and pro-oxidants (total fat, saturated fat, cholesterol, total carbohydrate, sucrose, and fructose) by group genotype. Only three differences (*p* < 0.05) in antioxidants consumption were observed which represent just the 4.2% of the total of possible combinations (*n* = 70) from 10 genotypes (two groups for each SNP) and 7 oligoelements studied. For the case of pro-oxidants, only seven differences (*p* < 0.05) were identified (11.6%) of the total of possible combination (*n* = 60) of 6 molecules and 10 genotypes reported.

On the other hand, a statistically significant (*p* < 0.05) improvement in the markers as weight (WG), body mass index (BMI), waist circumference (WC), GC, TG, TC, LDL-C, HDL-C, atherogenic index (AI), systolic (SBP), and diastolic blood pressure (DBP) was observed after 3 months of the trial in the participants; body fat (BF) and visceral fat (VF) did not show any statistical difference (Table 1).

**Table 1:** Effect of the 3-month dietary intervention on biochemical, clinical, and anthropometric variables on participants

Variables	Pre-intervention (*n* = 92)	Post-intervention (*n* = 92)	*p* value
Weight (kg)	91.9 ± 18.3 84.8	86.4 ± 15 84.4	< 0.031^a^
BMI (kg/m^2^)	34.9 ± 4.4 33.7	31.9 ± 4.25 30.4	< 0.001^a^
EI (Kcal/day)	1978 ± 326 1978	1926 ± 316 1840	0.320
Waist (cm)	96.5 ± 11.3 95.3	92.2 ± 10.2 92.3	< 0.001^b^
WHR	0.79 ± 0.14 0.81	0.80 ± 0.13 0.78	0.960
Body fat (%)	44.5 ± 7.6 42.8	40.8 ± 8.4 41.8	0.100
Visceral fat (cm^2^)	173 ± 47,147.9	157 ± 49,142.9	0.100
SBP (mgHg)	124 ± 9.7120	119 ± 12.5121	< 0.001^a^
DBP (mgHg)	86.1 ± 10.3 84	76.5 ± 7.9 76.5	< 0.001^a^
TBARS (mg/dl)	1.39 ± 0.57 1.31	1.03 ± 0.43 1.07	< 0.001^a^
Glucose (mg/dl)	123 ± 66.3101	108 ± 26.8 95.9	< 0.001^a^
Triglycerides (mg/dl)	154 ± 38.3156	151 ± 57.5150	0.003^a^
Total cholesterol (mg/dl)	177 ± 44.8163	154 ± 36.5152	< 0.001^a^
HDL-C (mg/dl)	42.4 ± 12.7 43	43.6 ± 12.2 43	0.400
LDL-C (mg/dl)	119 ± 48.1115.3	94.9 ± 35.7 88.4	< 0.001^a^
Atherogenic index	4.52 ± 1.73 4.19	3.96 ± 1.49 3.40	0.028^a^

Data show: *mean ± SD* median. *BMI* body mass index, *EI* daily energy intake, *WHR* waist–hip ratio, *SBP* systolic blood pressure, *DBP* diastolic blood pressure, *TBARS* thiobarbituric acid reactive substances, *HDL-C* high-density lipoprotein, *LDL-C* low-density lipoprotein

^a^Wilcoxon signed-ranks test was used to compare groups

^b^Paired *t* test was used to compare groups

### Response to nutritional intervention of participants stratified by genotype of CAT, GPX, and SOD

When the participants were stratified by CR and WT by each SNP studied, the results showed two patterns of response. The first observed pattern was a similar response of both groups (CR and WT), with respect to the changes observed at the end of the nutritional intervention for the SNPs 599C>T GPX1 (rs1050450), − 251A>G SOD1 (rs2070424), and − 262C>T CAT (rs1001179).

For the SNP 599C>T GPX1 (rs1050450), CR and WT showed statistical differences (*p* < 0.05) in five variables, BMI, SBP, TBARS, GLU, and LDL-C, when comparing the response before and after the dietary intervention within groups. In the case of the WT, they showed a statistically significant difference in the concentration of TG at the end of the intervention, while for CR, a statistically significant difference in AI and DBP was observed at the end of the intervention. A two-way ANOVA with replication analysis was performed (Table 2), finding a statistically significant interaction (*F* = 21.19; *p* < 0.001) between the gene polymorphisms and DBP.

**Table 2:** Effect of the dietary intervention on participants stratified by 599 C>T GPX1 (rs1050450) polymorphisms and interaction between period and group

599C>T GPX1 (rs1050450)						
					Interaction	
		CC (*n* = 36)	CT + TT (*n* = 56)	*p* ^1^	*F*	*p* ^2^
BMI (kg/m^2^)	Before	33 ± 5 31	35 ± 5 33	0.001	0.68	0.412
	After	30 ± 3 30^a**^	33 ± 4 32^a**^	0.003		
SBP (mgHg)	Before	120 ± 9120	124 ± 10,120	0.117	0.17	0.684
	After	114 ± 13120^a**^	119 ± 13120^a**^	0.049		
DBP (mgHg)	Before	85 ± 14 79	88 ± 8.9 88	0.375	21.19	< 0.001
	After	77 ± 8.4 75	79 ± 7.1 79^a**^	0.011		
TBARS (mg/dl)	Before	1.61 ± 0.61 1.40	1.34 ± 0.61 1.32	0.031	2.71	0.103
	After	1.08 ± 0.42 1.06^a**^	1.01 ± 0.45 1.02^a*^	0.611		
Glucose (mg/dl)	Before	127 ± 73,104	126 ± 68,105	0.600	0.79	0.374
	After	102 ± 29 99^a**^	105 ± 25 99^a**^	0.990		
Total cholesterol (mg/dl)	Before	176 ± 57,161	169 ± 34,169	0.489	1.11	0.295
	After	159 ± 43,152	150 ± 27 150^b**^	0.384		
LDL-C (mg/dl)	Before	117.9 ± 52.1107	126.5 ± 50.4125	0.174	0.01	0.914
	After	93.7 ± 35.8 89^a**^	102.6 ± 38.9 95.5^a**^	0.347		
Triglycerides (mg/dl)	Before	163.1 ± 32.3157	154.8 ± 39.2147	0.153	0.05	0.822
	After	161 ± 73.5 156^a*^	149 ± 48.7138	0.660		
Atherogenic index	Before	4.5 ± 3.3 4	4.1 ± 1.1 4.3	0.955	2.52	0.116
	After	3.8 ± 1.5 3.5	3.6 ± 1.4 3.3^a*^	0.033		

Data show: *mean ± SD* median, *BMI* body mass index, *SBP* systolic blood pressure, *DBP* diastolic blood pressure, *TBARS* thiobarbituric acid-reactive substances, *LDL-C* low-density lipoprotein. *p*^1^ *p* value of the comparison between groups (wild type and carriers) before and after the intervention. Mann-Whitney *U* test or two-sample *t* test was used to compare groups. *p*^2^ *p* value of the interaction. Interaction was calculated with two-way ANOVA with replication

^*^p < 0.05, ^**^p < 0.001

^a^Wilcoxon signed-rank test was used to compare between before and after the intervention in the same group

^b^Paired *t* test was used to compare between before and after the intervention in the same group

A similar pattern of response was observed in the SNP − 251A>G SOD1 (rs2070424), in which both CR and WT showed a statistically significant (*p* < 0.05) in seven variables BMI, SBP, DBP, TBARS, GC, TC, and LDL-C at the end of the nutritional intervention. Only a statistically significant interaction (*F* = 8.88; *p* = 0.004) was found between the gene polymorphisms and SBP (Table 3).

**Table 3:** Effect of the dietary intervention on participants stratified by − 251 A>G SOD1 (rs2070424) and 47C>T SOD2 (rs4880) polymorphisms, and interaction between period and group

		− 251A>G SOD1 (rs2070424)						47C>T SOD2 (rs4880)			
					Interaction					Interaction	
		AA (*n* = 53)	AG + GG (*n* = 39)	*p* ^1^	*F*	*p* ^2^	CC (*n* = 30)	TC + TT (*n* = 62)	*p* ^1^	*F*	*p* ^2^
BMI (kg/m^2^)	Before	34 ± 4.9 33	32 ± 4.5 30	0.021	0.32	0.570	35 ± 4.1 32	34 ± 4.8 33	0.047	105.37	< 0.001
	After	*33 ± 4.2 31* ^a**^	*31 ± 3.8 30* ^a**^	0.009			*32 ± 5 31* ^a*^	*32 ± 4.1 31* ^a**^	0.105		
SBP (mgHg)	Before	124 ± 9.8120	120 ± 8.9119	0.082	8.88	0.004	122 ± 12,121	123 ± 7.7121	0.736	4.66	0.034
	After	*117 ± 11120* ^a**^	*113 ± 13120* ^a*^	0.759			120 ± 13,120	*113 ± 12 120* ^a**^	0.594		
DBP (mgHg)	Before	85 ± 10 84	89 ± 9 90	0.008	2.23	0.139	85 ± 9.8 85	87 ± 10 85	0.461	0.05	0.824
	After	*78 ± 7.9 76* ^a**^	*77 ± 6.8 79* ^a**^	0.489			*78 ± 9.1 78* ^a**^	*77 ± 6.4 76* ^a**^	0.320		
TBARS (mg/dl)	Before	1.41 ± 0.64 1.23	1.48 ± 0.57 1.41	0.429	0.26	0.614	1.30 ± 0.42 1.20	1.42 ± 0.65 1.30	0.443	6.59	0.012
	After	*1.09 ± 0.42 1.02* ^a**^	*1.11 ± 0.5 1.09* ^a**^	0.446			1.18 ± 0.49 1.20	*1.00 ± 0.47 1.05* ^a**^	0.036		
Glucose (mg/dl)	Before	135 ± 82,105	104 ± 25,103	0.254	1.47	0.229	127 ± 80,101	127 ± 60,102	0.223	0.31	0.576
	After	*107 ± 32 100* ^a**^	*98 ± 9.5 97* ^a**^	0.040			*106 ± 32 92* ^a**^	*101 ± 19 96* ^a**^	0.009		
Total cholesterol (mg/dl)	Before	174 ± 51,166	173 ± 38,169	0.613	0.85	0.358	170 ± 35,164	174 ± 43,175	0.747	7.87	0.006
	After	*153 ± 40 146* ^a**^	*156 ± 30 153* ^a*^	0.265			156 ± 36,144	*154 ± 39 153* ^a**^	0.164		
LDL-C (mg/dl)	Before	118 ± 52,105	125 ± 41,122	0.275	0.00	0.954	108 ± 52 90	124 ± 43,123	0.066	10.95	0.001
	After	*93 ± 36 84* ^a**^	*94 ± 31 92* ^a**^	0.445			88 ± 32 79	*96 ± 35 94* ^a**^	0.527		
Triglycerides (mg/dl)	Before	161 ± 42,152	149 ± 26,148	0.068	3.53	0.064	167 ± 43,159	151 ± 36,148	0.127	0.00	0.961
	After	167 ± 69,158	*132 ± 43 122* ^a*^	0.001			168 ± 59,150	154 ± 59,146	0.148		
Atherogenic index	Before	4.7 ± 2.3 4.1	4.2 ± 1.0 4.2	0.853	3.30	0.073	4.4 ± 2.6 4.2	4.5 ± 1.6 4.1	0.543	2.49	0.118
	After	4.0 ± 1.5 3.5	3.9 ± 1.3 3.8	0.138			4.3 ± 1.7 3.6	*3.9 ± 1.4 3.4* ^a*^	0.074		

Data show: *mean ± SD* median, *BMI* body mass index, *SBP* systolic blood pressure, *DBP* diastolic blood pressure, *TBARS* thiobarbituric acid-reactive substances, *LDL-C* low-density lipoprotein. *p*^1^ *p* value of the comparison between groups (wild type and carriers) before and after the intervention. Mann-Whitney *U* test or two-sample *t* test was used to compare groups. *p*^2^ *p* value of the interaction. Interaction was calculated with two-way ANOVA with replication

^*^p < 0.05, ^**^p < 0.001

^a^Wilcoxon signed-rank test was used to compare between before and after the intervention in the same group

^b^Paired *t* test was used to compare between before and after the intervention in the same group

In the case of the polymorphism − 262C>T CAT (rs1001179), five variables showed a statistically significant difference between the beginning and the end of the dietary intervention in both CR and WT: BMI, SBP, DBP, TBARS, and GC. When the interaction between the gene polymorphisms and the variables of interest was analyzed using the two-way ANOVA with replication analysis, a statistically significant interaction was observed in three variables. The first statistically significant interaction was between the gene polymorphisms and SBP (*F* = 7.13; *p* = 0.009), in which CR and WT response showed a statistically significant difference (*p* < 0.05) at the end of the intervention. A statistically significant interaction was found between TC and gene polymorphisms (*F* = 4.37, *p* = 0.039), but only in the CR a statistically significant difference was observed at the end of the intervention. Finally, the LDL-C variable showed an interaction (*F* = 22.96, *p* < 0.001) with the gene polymorphisms, but only the WT showed a statistically significant difference at the end of the dietary intervention (Table 4).

**Table 4:** Effect of the dietary intervention on participants stratified by − 21A>T CAT (rs7943316) and − 262C>T CAT (rs1001179) polymorphisms, and interaction between period and group

		− 21A>T CAT (rs7943316)					− 262C>T CAT (rs1001179)				
					Interaction					Interaction	
		AA (*n* = 24)	TA + TT (*n* = 68)	*p* ^1^	*F*	*p* ^2^	CC (*n* = 55)	TC + TT (*n* = 37)	*p* ^1^	*F*	*p* ^2^
BMI (kg/m^2^)	Before	31 ± 2 30	36 ± 5 33	< 0.001	0.38	0.541	35 ± 3 33	35 ± 6 33	0.412	0.02	0.881
	After	*29 ± 2 28*^a^**	*34 ± 4 31*^a^**	< 0.001			*32 ± 3 30*^a^**	*33 ± 5 30*^a^**	0.119		
SBP (mgHg)	Before	122 ± 9120	121 ± 9.5120	0.992	4.48	0.037	123 ± 8.7122	119 ± 11,120	0.003	7.13	0.009
	After	120 ± 9.6120	*115 ± 13 119*^a^**	0.043			*119 ± 11 121*^a^**	*112 ± 14 119*^a^*	0.178		
DBP (mgHg)	Before	89 ± 11 85	85 ± 9.9 85	0.225	10.37	0.002	85 ± 10 85	87 ± 9.7 87.5	0.860	0.47	0.492
	After	*75 ± 8.4 77*^a^**	*78 ± 7.6 77*^a^**	0.186			*77 ± 5.8 75*^a^**	*78 ± 9.4 79*^a^**	0.320		
TBARS (mg/dl)	Before	1.45 ± 0.6 1.38	1.43 ± 0.5 1.27	0.894	2.55	0.114	1.42 ± 0.6 1.21	1.42 ± 0.5 1.46	0.343	0.54	0.463
	After	1.15 ± 0.5 1.27	*1.04 ± 0.4 1.03*^a^**	0.006			*1.3 ± 0.6 1.12*^a^*	*0.97 ± 0.2 1.05*^a^**	0.108		
Glucose (mg/dl)	Before	119 ± 26,105	129 ± 77,100	0.013	0.27	0.603	133 ± 80,104	111 ± 17,103	0.569	2.05	0.155
	After	*103 ± 9.1 98*^a^**	*106 ± 27 98*^a^**	0.363			*105 ± 30 96*^a^**	*96 ± 9.7 96*^a^**	0.231		
Total cholesterol (mg/dl)	Before	156.8 ± 37,155.5	177 ± 44,173.4	0.033	13.26	< 0.001	179 ± 51,178	165 ± 34,166	0.300	4.37	0.039
	After	155.1 ± 29,149	*155.8 ± 40,147.6*^a^**	0.615			163 ± 43,156	*145 ± 23 140*^a^**	< 0.001		
LDL-C (mg/dl)	Before	105.6 ± 38 98	125 ± 49,126	0.038	1.79	0.184	136 ± 46,124	99 ± 43 90	< 0.001	22.96	< 0.001
	After	*91.5 ± 32 83*^a^*	*96.4 ± 38 94*^a^**	0.100			*105 ± 37 95*^a^**	80 ± 35 78	0.240		
Atherogenic index	Before	4.4 ± 1.6 4.3	4.4 ± 1.8 4.5	0.350	5.35	0.023	4.8 ± 1.5 4.3	3.9 ± 1.3 4.4	0.297	1.62	0.206
	After	3.6 ± 1.5 3.8	*3.7 ± 1.4 3.1*^a^**	0.049			4.1 ± 1.7 4.5	3.7 ± 1.3 3.6	0.006		

Data show: *mean ± SD* median, *BMI* body mass index, *SBP* systolic blood pressure, *DBP* diastolic blood pressure, *TBARS* thiobarbituric acid reactive substances, *LDL* low density lipoprotein. *p*^1^ *p* value of the comparison between groups (wild type and carriers) before and after the intervention. Mann-Whitney *U* test or two-sample *t* test was used to compare groups. *p*^2^ *p* value of the interaction. Interaction was calculated with two-way ANOVA with replication

^*^p < 0.05, ^**^p < 0.001

^a^Wilcoxon signed-rank test was used to compare between before and after the intervention in the same group

^b^Paired *t* test was used to compare between before and after the intervention in the same group

The other pattern of change was related to the statistically significant difference in several clinical, anthropometric, and clinical variables between the beginning and the end of the dietary intervention only in CR, for the SNPs − 21A>T CAT (rs7943316) and 47C>T SOD2 (rs4880).

For the case of the SNP − 21A>T CAT (rs7943316) CR and WT showed a statistically significant difference at the end of the intervention in four variables BMI, DBP, GC, and LDL-C. When analyzing the interaction of the polymorphism with the aforementioned variables through the two-way ANOVA with replication, a statistically significant interaction was observed between the DBP variable and the gene polymorphisms (*F* = 10.37, *p* = 0.002). A statistically significant difference (*p* < 0.05) was observed only in the − 21A>T CAT (rs7943316) CR in three variables at the end of the nutritional intervention, in which a statistically significant interaction was found with the genetic polymorphism, SBP (*F* = 4.48, *p* = 0.037), TC (*F* = 13.26, *p* < 0.001), and AI (*F* = 5.35, *p* = 0.023) (Table 4).

For the case of the SNP 47C>T SOD2 (rs4880) CR and WT showed a statistically significant difference at the end of the intervention in three variables BMI, DBP, and GC. When analyzing the interaction of the gene polymorphisms with the aforementioned variables using the two-way ANOVA with replicates, a statistically significant interaction was found with the BMI variable (*F* = 105.37, *p* < 0.001). A statistically significant difference (*p* < 0.05) was observed in four variables in the CR at the end of the nutritional intervention. Those variables also showed a statistically significant interaction with the gene polymorphisms: SBP (*F* = 4.66, *p* = 0.034), TBARS (*F* = 6.59, *p* = 0.012), TC (*F* = 7.87, *p* = 0.006), and LDL-C (*F* = 10.95, *p* = 0.001) (Table 3).

In the multivariate analyses, the statistically significant differences observed between WT and CR of 47C>T SOD2 (rs4880) after the intervention were on IMC in model 4 (*p* = 0.029), on TBARS in model 1 (*p* = 0.023), model 3 (*p* = 0.017), and model 4 (*p* = 0.028); between WT and CR of − 21A>T CAT (rs7943316) were on DBP in model 2 (*p* = 0.001), model 3 (*p* = 0.002), and model 4 (*p* = 0.020).

## Discussion

The present work addresses the participation of five SNPs of the principal antioxidant enzymes, from a nutrigenetic point of view in people living with obesity. These five particular SNPs were selected due to their role in the etiology of obesity and its principal comorbidities, worldwide and in our population.

The work started with 153 mestizo Mexican women from the central plateau of Mexico; nonetheless, only 92 women finished the trial with an adherence equal or more than 90%. Several reasons account the desertion; however, two of these reasons (non-exclusive) were the principal ones: First, the inability of the participants to have an adequate adherence to the nutritional intervention at home, lack of support from their families to implement new healthy behaviors, and lack of possibility for the participants to develop two different menus due to cost and time. Secondly, the inability to perform the 30 min of daily physical activity due to a lack of time (work, chores, commute) or not having an adequate place to exercise (overcrowding or unsafe conditions). These findings on the cultural and environmental conflicts are not new; however, we wish to remark them because they are very important aspects present in people from low-income communities in developing countries like ours, which promote the obesogenic problem.

When we analyze the response of total of participants (*n* = 92) that finished the intervention, they showed good results in regard to the biochemical, anthropometric, and clinical variables studied. These results were obtained by means of a nutritional intervention together with an accessible physical activity recommendation (walk at a “brisk pace” by 30 min) since exercise has been demonstrated to improve the metabolic profile in people with obesity [32]. This improvement is directly associated with the reduction in body fat especially visceral fat since the visceral fat by means of proinflammation and oxidative stress conditions plays an important role in the appearance of obesity comorbidities as diabetes type II, resistance insulin, metabolic syndrome, dyslipidemia, etc. [33]. Furthermore, a body weight variation due to an energy-restricted diet in obese subject has been correlated with gene expression responses in processes of energy metabolism (oxidative phosphorylation, mitochondrion, generation of precursor metabolites and energy, regulation of lipid metabolism), focal adhesion and inflammation (T cell activation) in adipose tissue [34].

On the other hand, two kinds of responses were observed from the stratification of participant’s genotype and by evaluating their response by the end of the trail. The first one was identified as the capacity of the CR and the WT of SNPs 599C>T GPX1 (rs1050450), − 251A>G SOD1 (rs2070424), and − 262C>T CAT (rs1001179) to respond in a similar way after the nutritional intervention.

The second kind of response was the capacity of only the CR of SNPs − 21A>T CAT (rs7943316) and 47C>T SOD2 (rs4880) to get statistical changes in several variables at the end of the nutritional intervention. The response mentioned before of both SNPs by CR is notable, as the polymorphism − 21A>T CAT (rs7943316) showed a statistically significant interaction with four variables (SBP, DBP, TC, and AI), while the polymorphism 47C>T SOD2 (rs4880) showed a statistically significant interaction with five variables (BMI, SBP, TBARS, TC, and AI)**.** Two of these last variables were common for both polymorphisms (SBP and TC).

We expected that the response of the WT people would be better in general in comparison with mutated CR, but to our surprise, none of the WT groups of the five SNPs displayed this characteristic. In this respect, evidence [35–38] has shown that the mutated allele of several SNPs studied in the present work was associated with more severe complications or comorbidities in people with metabolic disease as diabetes type I and type II. In particular, Ascencio-Montiel et al. [39] identified an association between macroalbuminuria (as a predictor for diabetic nephropathy) in Mexican people with diabetes type II and the homozygous mutated TT genotype of 47C>T SOD2 (rs4880).

We do not have an extended explanation related to the superior response shown by CR of SNPs − 21A>T CAT (rs7943316) and 47C>T SOD2 (rs4880). However, CAT is a relevant ubiquitous enzyme that neutralizes the hydrogen peroxide, it is a principal antioxidant cytoplasmic enzyme, and it seems to play an important role in the obesogenic process and in the developments of its comorbidities.

At the functional level, a previous report identified a significant inverse correlation between the activity of plasma erythrocyte enzyme with weight, BMI, plasma insulin, and HOMA in a case study (*n* = 194) and controls (*n* = 191) among Spanish children with obesity [25]. In the same study, the − 21A>T CAT (rs7943316) polymorphism was associated with an increased risk for the development of obesity (OR = 1.38, CI 95%, 1–1.91; *p* < 0.05) as well as the neighbor polymorphisms − 844A>G CAT (rs769214) and − 20C>T CAT (rs1049982).

In the molecular field, Saify [40] described for the first time the appearance of a nuclear binding site for the nuclear transcriptional factor PAX-6, when the substitution of adenine to thymine is present in the SNP − 21A>T CAT (rs7943316). The PAX-6 is a highly conserved multifunctional transcription factor that takes part actively in the development of the eye and brain, and it has been proposed to bind to the promoter region of CAT gen to regulate its expression. Directly in line with previous data mentioned, Saify et al. [41] identified the mRNA gene expression of CAT from peripheral mononuclear cells stratified by AA, AT, and TT genotype of − 21A>T CAT (rs7943316) polymorphism. A significant positive correlation was observed between the mRNA level expression of CAT by mononuclear cells and the presence of thymine allele. Thus, a higher antioxidant CAT enzyme capacity could be involved in the better response shown by participant CR of T allele in our study.

For the case of 47C>T SOD2 (rs4880), the argument of a higher enzyme capacity is not possible because of the presence of the thymine allele in CR of this SNP, abrogates the enzymatic activity of SOD2 around 30–40% [21]. Though, inconsistent results have been shown related to the interaction between the SNP 47C>T SOD2 (rs4880) and antioxidant status; likewise, dietary factors can modulate its activity [15, 42].

A multivariate analysis adjusted for different variables was carried out with the aim to observe the effect of the SNPs of interest in the present study on the identified response at the end of the study in the participants (overlapping) as well as other variables such as age and BMI. We identified that in general, there is not relevant overlapping effect since in the case of − 21A>T CAT (rs7943316), in which we identified a statistically significant interaction in four variables between CR and WT at the end of the intervention (SBP, DBP, TC, AI), we observed a statistically significant value only on DBP in the adjusted models 2, 3, and 4. While in the case of 47C>T SOD2 (rs4880), we identified a statistically significant value in BMI, in the adjusted model 4, and in TBARS, in the adjusted models 1, 3, and 4.

In the specific case of model 1, where we evaluate the participation of the other polymorphism showing a statistically significant interaction in the two-way ANOVA with replication analysis (− 21A>T CAT or 47C>T SOD), we only identified a statistically significant difference between CR and WT in TBARS. This result seems consistent with physiological oxidation-reduction process that the study participants presented at the end of the study since the aforementioned polymorphisms actively participate in the regulation of the cellular redox process.

To our knowledge, this is the first work related to the influence of the five SNPs studied herein on a nutrigenetic study in people with obesity. The limitation of a relatively small number of participants that finished the study is compensated by the homogeneous group of subjects formed. People who finished the intervention showed a high adherence to the nutritional and physical activity maneuver, and they were a non-related mestizo genetic group from a typical low-incoming community of central plateau of Mexico, with fathers and grandfathers born in Mexico.

Although diverse SNPs have been associated worldwide with several metabolic diseases, it is necessary to increase this knowledge on each population because every ethnic group has a specific set of genes implicated in these pathologies, as a result of the own “gene-imprinting” and “gene-transfer,” diet, geographic area of habitat, lifestyle, etc. [43, 44].

On the other hand, even if there is a great probability that several SNPs are involved in the observed final physiological response in subjects under a dietary intervention, the SNPs − 21A>T CAT (rs7943316) and 47C>T SOD2 (rs4880) reported in the present work could be used in future studies as a genetic tool to improve the treatment of overweight and obesity as well as to identify, trace, and evaluate the risk to develop several comorbidities of obesity.

## Conclusion

The CR of SNPs − 21A>T CAT (rs7943316) and 47C>T SOD2 (rs4880) are implicated in a better response in anthropometric, clinical, and biochemical markers in comparison with their respective WT group after a nutritional intervention by 3 months. However, the participation of other SNPs related to enzymatic antioxidant defense can be implicated on the results of the present study.

## Abbreviations

AI: Atherogenic index
AV: Anthropometric variables
BMI: Body mass index
BP: Body fat
BV: Biochemical
CAT: Catalase
HDL-C: High-density lipoprotein cholesterol
LDL-C: Low-density lipoprotein cholesterol
CR: Carriers
CV: Clinical variables
DBP: Diastolic blood pressure
GC: Glucose
GPX1: Glutathione peroxidase
SBP: Systolic blood pressure
SNPs: Single nucleotide polymorphisms
SOD: Superoxide dismutase
TBARS: Thiobarbituric acid reactive substances
TC: Total-cholesterol
TG: Triglycerides
VF: Visceral fat
WC: Waist circumference
WG: Weight
WT: Homozygous wild type
